# A Local and Non-Local Features Based Feedback Network on Super-Resolution

**DOI:** 10.3390/s22249604

**Published:** 2022-12-07

**Authors:** Yuhao Liu, Zhenzhong Chu, Bin Li

**Affiliations:** 1 College of Information Engineering, Shanghai Maritime University, Shanghai 201306, China; 2 School of Mechanical Engineering, University of Shanghai for Science and Technology, Shanghai 200093, China; 3 School of Computer Science, Northeast Electric Power University, Jilin 132012, China

**Keywords:** single-image super-resolution, non-local self-attention, feedback network, deep convolutional network, dense skip block

## Abstract

Recent advances in Single Image Super-Resolution (SISR) achieved a powerful reconstruction performance. The CNN-based network (both sequential-based and feedback-based) performs well in local features, while the self-attention-based network performs well in non-local features. However, single block cannot always perform well due to the realistic images always with multiple kinds of features. In order to take full advantage of different blocks on different features. We have chosen three different blocks cooperating to extract different kinds of features. Addressing this problem, in this paper, we propose a new Local and non-local features-based feedback network for SR (LNFSR): (1) The traditional deep convolutional network block is used to extract the local non-feedbackable information directly and non-local non-feedbackable information (needs to cooperate with other blocks). (2) The dense skip-based feedback block is use to extract local feedbackable information. (3) The non-local self-attention block is used to extract non-local feedbackable information and the based LR feature information. We also introduced the feature up-fusion-delivery blocks to help the features be delivered to the right block at the end of each iteration. Experiments show our proposed LNFSR can extract different kinds of feature maps by different blocks and outperform other state-of-the-art algorithms.

## 1. Introduction

Single-Image Super-Resolution (SISR) aims to reconstruct a High-Resolution (HR) image from a single Low-Resolution (LR) input image. The mapping between LR and HR images is not bijective, leading to an ill-posed problem. The recent success of image SR can greatly enhance the quality of the generated image result [1]. There are two typical SISR methods. (1) The traditional methods, which focus on improving the performance with better PSNR, such as deep convolutional networks [2], residual networks [3], feedback networks [4] and self-attention [5] based HR. (2) The GAN-based SR, such as SRGAN [6], is another way to generate SR image. GAN-based SR can generate ‘look more like natural’ SR image [7] by introducing a discriminator network to direct the generator network to generate more real SR images, but maybe not performing better on PSNR. In this paper we focus on the traditional methods, improving the performance with better PSNR.

Most of the state-of-the-art SISR algorithms assume all the HR images own similar features and propose an ingenious design block to extract them, which is not suitable for all the realistic images, due to the realistic images always having different kinds of features (such as Local and non-local features, feedbackable and non-feedbackable features). Generally: Feedback-based SR assumes the padding detail information is repeatable and can be extracted from the same network with the same weight value iteration by iteration; the deep convolutional network assumes the padding detail can be extracted from the nearby feature of input images (receptive field is limited by kernel size), but failed to make use of faraway features; self-attention-based SR scoring different features, assuming the padding detail is composed of higher scoring features, but the discarded low score value features may be critical for a small amount of image realistically.

Addressing the above shortage, we separate the SR images into five import parts: (1) The based LR features, which are considered as part of the output by up-sampling to the desired size. (2) The local feedbackable features, which are within the kernel-size region of image pixels and feedbackable. (3) Local non-feedbackable features which are unique and local, these kinds of features are always dropped for most Feedback-based algorithms, but we keep them. (4) Non-local feedbackable features, which are a distance from each other and feedbackable. (5) Non-local non-feedbackable features which are a distance from each other but unique and non-feedbackable features, this part of the features is always dropped for most self-attention-based and feedback-based algorithms.

Based on the above analysis, in this paper, we choose different network blocks to extract the five features mentioned above from LR images: (1) The residual network is a simple but powerful skill, so we take the residual connection to deliver the based LR features, then up-sample and compose with residual features to HR images. (2) The non-local feedbackable features can be extracted by self-attention, which can extract the distance feature-patch, and take use of the distance feature-patch to remedy the loss of feature details. (3) The convolutional-based feedback network can extract the local feedbackable features efficiently. (4) The traditional deep convolutional network can extract the local non-feedbackable features easily. In our proposed algorithm, the deep convolutional network is considered as a compensation of other blocks and only needs to extract the minority non-feedbackable features. (5) We choose a reusable strategy, which will reduce the network scale, to extract the non-local non-feedbackable features.

How to take full use of the above-mentioned features is critical for the network performance, simply concatenating all the blocks together will reduce the performance. So, in this paper, we also proposed the up-fusion-delivery block to merge the features together and then deliver different features to the right block quickly.

In summary, the main contributions of this paper are two-fold:

1. We separate the SR images into five parts and choose three famous blocks (VDSR [3] block, SRFBN [4] block and CSNL [5] block) to generate the corresponding SR image parts. Because each block is designed to work on what they do best, this structure can take full use of each block’s advantage and compensate for its shortage with other blocks.
2. We proposed the up-fusion-delivery block at the end of each iteration, which will help the feature be delivered to the right block on the next iteration. Features can be extracted and delivered to the right block quickly, so our LNFSR algorithm only needs fewer iterations to achieve comparable performance (6 for our LNFSR, while 12 for CSNLN).

The experiments show our proposed LNFSR outperforms other state-of-the-art algorithms.

## 2. Related Works

In this section, we will give a brief introduction to deep convolutional-based, residual connection-based, feedback-based and self-attention-based SR, all are the basic methods of our proposed SR method.

SRCNN [2] is well-known as the first deep convolutional layers-based SR algorithm. VDSR [3] is another deep convolutional-based SR, the VDSR is very deep with 20 convolutional layers and 19 ReLU layers between them. The VDSR shows that a deeper network will improve the SR performance, but it is difficult to converge, so residuals connection and gradient clipping skills are introduced. Both SRCNN and VDSR are classical algorithms, the performances are not superior, but are still an inspiration for other algorithms.

The Residual Connection-based SR, the SR image is added by its input LR enlarged with the network output [8], is critical for SR algorithms. The residual connection delivers the LR image directly to the SR image, so the network only needs to compute the residual information, which reduces the burden of the network and is easy to converge, so the network can go very deep. Lim et al. [9] introduced an Enhanced Deep Super-Resolution Network for SR (EDSR) based on SRResNet [6] architecture, they first optimized the network by removing unnecessary modules, so there are only two convolutional layers with ReLU between them. They also proposed a multi-scale deep SR network (MDSR) that can effectively deal with various scales (×2, ×3 and ×4) of super-resolution in a unified framework to reduce the model size and training time. The EDSR is powerful in local features but it failed to extract non-local features, and too many layers of the sequential stack will lead to large network parameters.

There are extensively studied on the Feedback-based SR. Kim et al. [10] proposed a Deeply-Recursive Convolutional Network (DRCN) for SR, the DRCN network has a very deep recursive layer (16 recursions), the deeper recursive layer can extend feature extraction area without introducing the number of parameters. Liu et al. [11] introduced the Residual Feature Aggregation (RFA) framework which groups several residual modules output together, so different levels of residual modules (considered as the hierarchical features on the residual branches) can take fully used. Li et al. [4] proposed SRFBN which is a feedback mechanism based on dense connections. The base block, which is the deconvolutional layer that follows a convolutional layer, can enlarge the feature maps with the deconvolutional layer and then get rid of useless information with the convolutional layer. The experiments demonstrate that SRFBN outperforms other selected methods. The feedback-based SR algorithm’s performance is excellent, especially in local features with small network scales, but they failed to extract non-local similar features, which is used to further improve the SR performance.

Self-attention, which plays an important role in human perception, has been studied extensively in the previous research [12,13]. Due to the self-similarity of images, in reality, small patches tend to recur within the same image under different scales. In order to use these features, Mei et al. [5] proposed the Cross-Scale Non-Local (CSNL) attention module. The CSNL model can extract non-Local features, even if features with different scales. The CSNL algorithm achieved the best performances in 2020, but they were costly due to the quadratic computational cost of the input size. Addressing this problem, Xia et al. [14] introduced sparse into the module and proposed a novel Efficient Non-Local Contrastive Attention (ENLCA) into SR. The ENLCA achieves comparable performance as the standard non-local module while merely requiring linear computation and space complexity with respect to the input size. However, the ENLCA did not consider the different scale’s similar features, which will limit the performance of the ENLCA-based SR algorithms.

All the blocks mentioned above have their own advantages: the deep convolutional-based SR focus on extracting local unique feature, the feedback-based SR focuses on repeating refined local features, self-attention focuses on extracting non-local features, and the residual connection is a powerful skill to reduce the burden of the network. All algorithms mentioned above performed best at the right time but failed to cooperate with other algorithms to composite their shortages. So, in this paper, we take the divide-and-conquer strategy, making different blocks work on what they do best.

## 3. Local and Non-Local Feature Based Feedback Network for SR (LNFSR)

In this section, we introduce the proposed local and non-local feature-based feedback network for SR (LNFSR). First, we will introduce the network structure of the LNFSR in Section 3.1, then we will give a detailed description of the local and non-local feature extraction block structure in Section 3.2 and Section 3.3. Last, we will discuss the implementation details not mentioned above in Section 3.4. The acronyms and notations used in this Section are listed in Appendix A (Table A1).

### 3.1. The Network Architecture of our LNFSR

**Figure 1:**
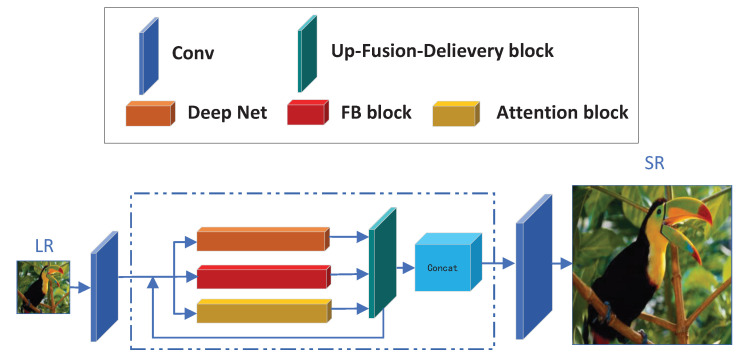
The Network Architecture of our LNFSR.

Our LNFSR mainly consists of three parts: The front feature extraction block, the local and non-local feature extraction block and the reconstruction block. Let us denote $I_{LR}$ as the input low-resolution image, $I_{HR}$ as the corresponding high-resolution image, and $I_{SR}$ as the super-resolution image which is the output of LNFSR. We give a detailed introduction to the network architecture of the LNFSR as Figure 1.

**Front Feature extraction block (FF block)**: The front feature extraction block is simply two convolutional layers denoted as FF(·), so the input of the FF block is the LR image ($I_{LR}$) while the output of the FF block $F_{FF}$ is:

(1) $$F_{FF}=FF\left(I_{LR}\right)$$

The FF block is simple but useful: 1. It extracts the shallow features to feed into the following block. 2. It extends the feature maps into the desired amount. 3. It generates the base LR features and basic-residual feature maps for the following blocks.

**Local and Non-local Feature extraction block (LNF block)**: The local and non-local feature extraction block is severed as the main feature extraction block of our LNFSR. The base LNF block is a feedback mechanism, during each iteration, the current iteration output of the feedback block is considered as the input of the next iteration. The outputs of all iterations are concatenated into large feature maps, this is the final output of the LNF block. The LNF block is denoted as Lnf(·), so the input of the LNF block is the output of the FF block ($F_{FF}$) while the output of the LNF block $F_{Lnf}$ is:

(2) $$F_{Lnf}=Lnf\left(F_{FF}\right)$$

We will give a detailed description of the LNF block in Section 3.2 and Section 3.3. The output of the LNF block is the up-scaled feature maps, which can be fed into the Reconstruction block to assemble the despaired SR output directly.

**Reconstruction block (Rb block)**: The reconstruction block is simply one convolutional layer, which reconstructs the SR image by assembling the feature maps of the LNF block’s output. So, the input of the Rb block is the output of the LNF block ($F_{Lnf}$), while the output of the Rb block is the final SR image ($I_{SR}$), denotes as:

(3) $$I_{SR}=Rb\left(F_{Lnf}\right)$$

The Rb block is only one convolutional layer which can reduce the vanishing gradient problem. Due to the LNF block having the most parameters, the gradient needs to be delivered to the LNF block quickly during the back-propagation process, so one convolution layer-based Rb block is desired.

**The Loss Function**: The LNFSR is optimized to minimize the loss function L(Θ), we choose the $L_{1}$ loss function which is the same as most of the previous works. Given a training image pair <$I_{LR}$, $I_{HR}$>, the loss function is defined as:

(4) $$L\left(\Theta \right)={∥LNFSR\left(I_{LR}\right)-I_{HR}∥}_{1}$$

where:

(5) $$LNFSR\left(I_{LR}\right)=I_{SR}=Rb\left(Lnf\left(FF\left(I_{LR}\right)\right)\right)$$

### 3.2. The Local and Non-Local Feature Extraction Block

**Figure 2:**
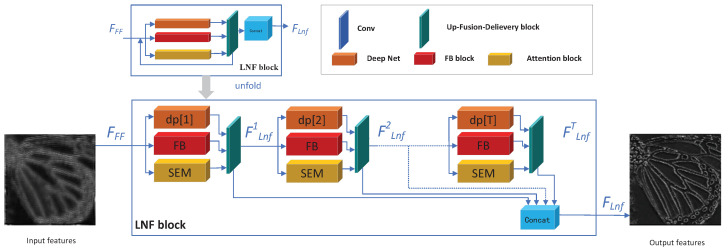
The Local and Non-local Feature extraction block.

In this section, we give a detailed introduction to our proposed local and non-local feature extraction block (LNF block). The structure of the proposed LNF block is described in Figure 2.

Figure 2 gives us a brief illustration of our proposed local and non-local feature extraction block (LNF block). The LNF block is a feedback-based network block, so the LNF block is *T* iterations, during each iteration (such as the *i*-th iteration), the previous iteration output ($F_{Lnf}^{i-1}$ if 1<i<n−1 and $F_{FF}$ if i=1) is considered as the input of *i*-th iteration’s, while the output of current iteration is $F_{Lnf}^{i}$. The outputs of all iterations ($F_{Lnf}^{1}$, $F_{Lnf}^{2}$, ⋯, $F_{Lnf}^{T}$) are concatenated as the output of the LNF block. The output of the LNF block is defined as:

(6) $$F_{Lnf}=concat\left(F_{Lnf}^{1},F_{Lnf}^{2},\cdots ,F_{Lnf}^{T}\right)$$

where the function concat(·) is to concatenate all the inputs on the feature dimension (second dimension).

We take all the feature maps as outputs during all iterations, due to different iterations being able to extract different levels of feature maps. We aggregate all the hierarchical features as [11] during all iterations by concatenating them together, so the following blocks (the Rb block) can take full use of different hierarchical features to assemble the desired SR images.

**The blocks on each iteration**: For each iteration (take the *i*-th iteration as an example), there are three basic blocks: the DP[*i*] block, the FB block, the CSNL block, the outputs of the three blocks are feed into one fusion layer as the output of current iteration ($F_{Lnf}^{i}$) and the next iteration’s input, as shown in Figure 2.

We choose our DP block as the famous VDSR [3], which is a simple but effective SR algorithm. Due to the feedback-based LNF block, the DP block needs to extract different feature maps during different iterations. To address this issue, we separate the DP network into *T* segments (DP[1], …DP[*T*]), so we only make use of the *i*-th part (denoted as DP[*i*]) of DP on the *i*-th iteration. From another perspective, the *T* segments of the DP network focus on different level features and flexible exchange features during different iterations.

We choose our FB block from Li et al.’s work [4], which is a well-designed feedback block. Li’s FB block can generate powerful high-level representations under the feedback scheme, which is *G* groups stacks, for each group, the convolutional layer and deconvolutional layer are connected, respectively. All the layers are densely connected. The deconvolutional following the convolutional connection can (1) enlarge the features by the deconvolutional layer so useful features can be emphasized, (2) compress the features with the convolutional layer so useless features are dropped. After *G* groups, the useless can be discarded and useful features can be highlighted.

We choose our CSNL block from Mei et al.’s work [5], which proposed a well-designed cross-scale non-local attention block. Mei’s CSNL block can extract non-local features by attention scheme under different scales. The CSNL block has two different non-local attentions: In-scale attention and cross-scale attention, both attention blocks can explore self-exemplars by summarizing related features from the whole image. The in-scale attention is a pixel-wise correlation with features, while the cross-scale non-local attention is a patch-wise correlation with features. We choose the CSNL block as our non-local feature extraction block due to its remarkable non-local feature extraction ability even if distance similar features under different scales.

In summary, we separate the SR image residual feature maps into five features and choose three famous blocks to extract them. The three paths are as follows: (1) Local feedbackable features: the FB block is taken to extract local repeatable features. (2) Non-local feedbackable features: the CSNL block is taken to extract the non-local repeatable features. (3) Local non-feedbackable features: the DP block is taken to extract the local non-repeatable features.

We did not choose five distinguish blocks to extract them due to reducing the scale of the LNF block. The analysis is as follows:

**(4)**  **Drop the based LR features**: We drop the skip connection from input to the end for the based LR features, due to there being skip connection insider the CSNL block [5], which plays the same row as the skip connection directly. Experiments show that adding an additional skip connection will drop the performance, the reason we guess is the LR features play a critical role for the CSNL block to extract non-local features and the CSNL block has its own skip connection which serves the same work, so we drop the outer skip connection.
**(5)**  **Drop the Non-local non-feedbackable features**: We did not take another distinct block to extract the non-local non-feedbackable features, due to the reuse strategy. Because the non-local non-feedbackable features are similar to the local non-feedbackable features but far away, the non-local non-feedbackable features can be extracted by the CSNL block and then delivered into the DP block to refine them. With the help of our proposed up-fusion-delivery layers during each iteration, features can be delivered to the right block on the next iteration.

### 3.3. The Up-Fusion-Delivery Layers of Our LNFSR

**Figure 3:**
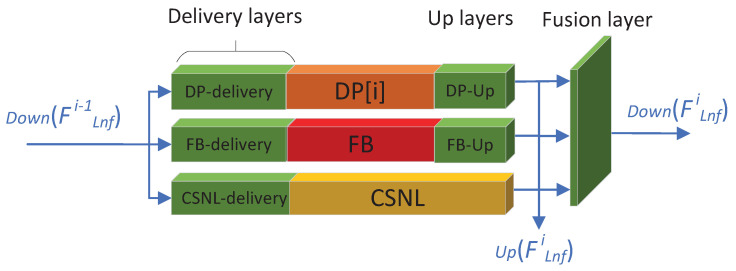
The detail of the up-fusion-delivery LNF block under the i-th iteration (**green block** denote the up-fusion-delivery layers).

We proposed the feature up-fusion-delivery strategy to combine three different blocks together, which serve a critical function in our proposed algorithms. Figure 3 is the detail of the LNF block under the *i*-th iteration, from Figure 3 we can see that there are Up layers, fusion layer and delivery layers on both sides of the DP[*i*] block, FB block and CSNL block.

**Up layers**: There are two up-layer blocks followed by DP[*i*] and FB block, up-sampling the outputs of DP[*i*] and FB block to desired up-scale (the CSNL block can generate desired up-scale output, so need not up layer block). The up layer is simply one convolutional layer following PReLU [15], the outputs of DP-Up, FB-Up and CSNL are concatenated, denoted as $Up(F_{Lnf}^{i})$, which is considered as the output of the LNF block under the *i*-th iteration, so the output of LNF block (Equation (6)) can also be denoted as:

(7) $$F_{Lnf}=concat\left(Up\left(F_{Lnf}^{1}\right),\cdots ,Up\left(F_{Lnf}^{T}\right)\right)$$

The $F_{Lnf}$ is the desired upscale feature map which can be assembled to SR directly. We generated the up-scale feature maps at the end of the LNF block during each iteration, because this strategy can balance the computation cost and fully use the middle feature information.

**Fusion layer**: The fusion layer is simply two convolutional layers following PReLU, it serves two functions: (1) It fuses three different kinds of feature maps together, so all the features can be fused and delivered to where they should go during next iteration. (2) It downscales the $Up(F_{Lnf}^{i})$ to the original input LR scale as the input of the next iteration, denoted as $Down(F_{Lnf}^{i})$, to reduce the computation cost.

**Delivery layers**: There are three different delivery layers ahead of the DP[*i*] block, FB block and CSNL block. They deliver the desired feature maps to the right block: the DP-delivery layer delivers the local non-feedbackable features to the DP[*i*] block, the FB-delivery layer delivers the local feedbackable features to the FB block, the CSNL-delivery layer delivers the non-local non-feedbackable features to the CSNL block, and the basic LR features are also delivered by the inner skip-connection of the CSNL block to the end. Each of the three delivery layers is only one convolutional layer that follows PReLU.

The up layer follows the fusion layers, the advantages are: (1) The up layer can focus on generating desired output of the current iteration, which fully uses the middle features information for SR and reduces the burden of the following Rb block. (2) The fusion layer reduces the feature maps scale into the LR scale, as the input of the next iteration, which can reduce the computational cost of the LNF block. (3) The up-fusion strategy can enlarge the useful features in the up process, then compress features leaving useful features for the next iteration in the down process.

**Figure 4:**
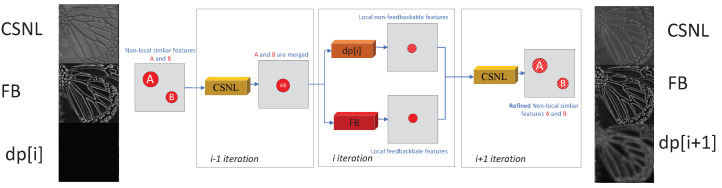
The non-local non-repeatable features’ extraction (hypothesis) process.

The fusion layer follows three Delivery layers, the advantages are: (1) The fusion layer focuses on refining features and preparing the desired feature maps for the next iteration, while three delivery layers focus on picking the right features for the right blocks. (2) The fusion-delivery strategy can take full use of the feature maps under different iterations, one feature can be delivered to different blocks in the current iteration, while many small features can reassemble as one feature in the next iteration. Figure 4 is one hypothesis on how the non-local non-feedback features’ extraction process under the fusion-delivery layers.

(8) $$\begin{array}{cc} F_{Lnf}^{i}=Fusion[ & DP_{up}\left(DP\left[i\right]\left(DP_{dlr}\left(F_{FF}\right)\right)\right),FBup\left(FB\left(FB_{dlr}\left(F_{FF}\right)\right)\right),\\ \; & CSNL(CSNL_{dlr}\left(F_{FF}\right))\;] \end{array}$$

In summary, the output of the *i*-th iteration $F_{Lnf}^{i}$ is defined as Equation (8). Where i∈[1,⋯,T], and the function $DP_{up}$, $FB_{up}$ is the up layer of the DP block and the FB block, the Fusion(⋯) is the fusion layer, the $DP_{dlr}\left(\cdot \right)$, $FB_{dlr}\left(\cdot \right)$ and $CSNL_{dlr}\left(\cdot \right)$ is the delivery layers, FB is the feedback block, and CSNL is the CSNL block, both FB and CSNL are feedback block which is repeated during all iterations, while the DP[i] block is the *i*-th part of the VDSR.

**Figure 5:**
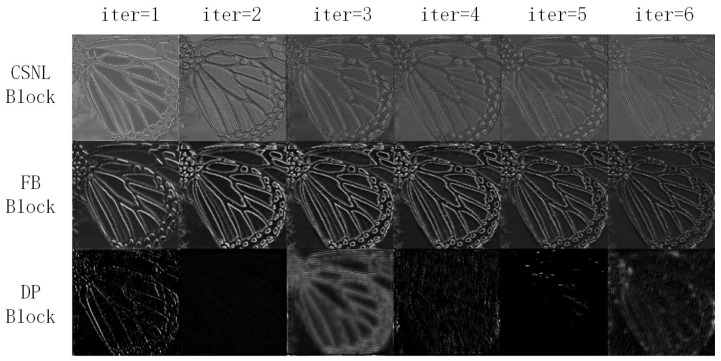
The outputs of 3 different blocks during all iterations under a single channel for our LNFSR.

**Hypothesis and Visualized analysis of the up-fusion-delivery layer**: Due to the difficulty analyzing the data transfer process obviously, we give one hypothesis in Figure 4 about the data transfer process, and a visualized analysis in Figure 5 to confirm our hypothesis un-rigorously.

Figure 4 gives us a hypothesis on the non-local non-feedbackable features’ extraction process of the proposed LNF block, we can see that: (1) The non-local similar features A and B, which are similar to each other under different scales, are far distance in a feature map at the very beginning, then (during the i−1 iteration) the feature map is fed into the CSNL block which can extract the non-local features roughly. (2) The features A and B are merged into one feature map due to similarity, then the feature map is fed into the DP[*i*] block and FB block (during the *i* iteration). (3) The DP[*i*] block focus on extracting the local non-feedback features while the FB block focuses on extracting the local feedback features. (4) (During the i+1 iteration), the non-local non-feedback features are disassembled and delivered to the original positions.

Figure 5 is a visualized analysis, which shows the outputs of three different blocks during six iterations under a single channel for our LNFSR. We can see that different block focus on different kinds of features: (1) Both the CSNL and FB block focus on feedbackable features, but slightly different styles. The CSNL block focuses on the skeleton-like feature, which is generated from a far distance, while the FB block focuses on a detail-like feature, which is generated locally. (2) The DP block generates non-feedbackable features, which are unique and have huge differences on different iterations, to refine the SR image. For the DP block’s feature maps, the output feature maps on both ends (iteration 1 and 6) are in the right place (butterfly-like feature maps), while the output feature maps on the middle position feature maps (iteration 2 to 5, especially iteration 4 and 5) are a blur and not in the right place. This confirms our hypothesis in Figure 4. Due to the merge process, the features are in an unpredictable position (iteration 2 to 5), then fed back to the right place in the last iteration.

### 3.4. Other Implementation Details

Following are the other implementation details not mentioned above:

(1) We follow the implementation details of the FB block and CSNL block. We use the ReLU as the activation function for DP blocks and PReLU as the activation function for other blocks.
(2) We set all the feature channels as 64 for all three blocks (DP block, FB block and CSNL block). Due to half channels for CSNL inner block, the CSNL input feature channel is 128 then half to 64 for the inner block of CSNL. We set the number of feedback iterations for the LNF block as 6 to balance the performance and cost. Our LNFSR has more parameters than CSNLN, due to adding the DP and FB block, there are 3.06 M (×2) and 6.57 M (×4) parameters for CSNLN, while 11.30 M (×2) and 18.91 M (×4) parameters for our LNFSR. However, by half the iterations (12 for CSNLN), the computational cost of our LNFSR is close to that of CSNLN. For one training iteration, it cost about 16 min (×2) and 140 min (×4) for CSNLN, while 18 min (×2) and 93 min (×4) for our LNFSR on single NVIDIA 3090 GPU.
(3) We choose $L_{1}$ loss to optimize our LNFSR, the Adam optimizer to optimize the parameters of the network with β1=0.9, β2=0.999 and the initial learning rate 0.0001, we reduce the learning rate by multiplying 0.5 for every 200 epochs for a total of 1000 epochs. The network is implemented with the PyTorch framework and trained on a single NVIDIA 3090 GPU.

## 4. Experimental Results

### 4.1. Datasets and Evaluation Metrics

For training, we perform all the experiments on the DIV2K database (total of 1000 images, where 800 images as the training set, 100 images as a valid set and 100 images as the test set), we take all the train set (800 images) to train our models. All the experiments are performed in 1000 iterations. The LR images generated by taking the BiCubic method from HR images, each LR image is random cropped into one small patch (We set the input patch size =48×48 to balance the performance and cost for our LNFSR) as the input LR images. We also perform image reuse and augmented strategy: During each iteration, all the train images are performed 10 times and the <$I_{LR}$,$I_{HR}$> image pairs are augmented by random rotating $90^{{}^{\circ}}$, $180^{{}^{\circ}}$, $270^{{}^{\circ}}$ and horizontal flipping. We report the performance on four famous standard benchmark datasets: Set5 [16], Set14 [17], B100 [18] and Urban100 [19]. We evaluated all the SR results on PSNR and SSIM [20].

### 4.2. Ablation Study

In this Section, we will perform an ablation study on our proposed LNFSR. In order to reduce the training cost, we set the feature channel as 32, feedback iterations as 4 and iteration times as 500, and the learning rate decay for every 150 epochs, which is denoted as LNFSR-L. The ablation study focuses on our two contributions: (1) Whether the local and non-local feature extraction blocks outperform the single blocks? (2) Whether the up-fusion-Delivery block help to improve the performance?

**Ablation study on the three blocks in our LNF block:** We perform an ablation study to determine whether the three blocks in our LNF block will improve the performance. We keep only one block and drop the other two blocks in the LNF as comparing algorithms. The Only-DP denotes the algorithm with only the DP block (VDSR block), the Only-FB denotes the algorithm with only the FB block, and the Only-CSNL denotes the algorithm with only the CSNL block. All the comparing algorithms with the same training parameters. The results are listed in Table 1. Our proposed LNFSR-L outperformed all the comparing algorithms, in Table 1, illustrating that the local and non-local feature extraction blocks outperform the single block structures.

**Figure 6:**
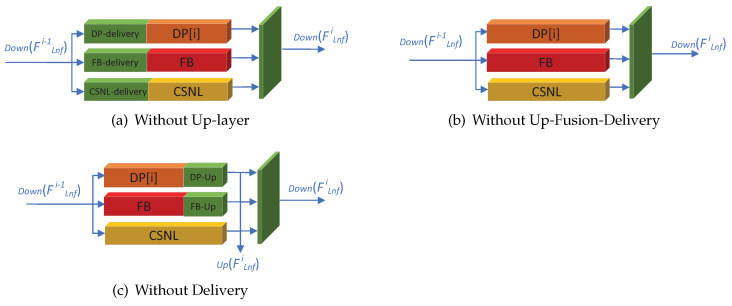
The 3 different structures for the ablation study on the up-fusion-delivery block.

**Ablation study on up-fusion-delivery block:** We perform an ablation study to determine whether the up-fusion-Delivery will improve the performance. We designed three typical fusion methods with different structures to fuse three blocks together, as shown in Figure 6. Without Up-layer: we drop the up layer, so the output of three blocks, without the up-scales process, is fed to the fusion layer as the input of the next iteration. Without Delivery: we drop the delivery layer for three blocks, keeping the up-fusion block, so the input is fed directly into three blocks. Without up-fusion-delivery: we drop all the up-fusion-delivery blocks, so the output of three blocks is fed directly into three blocks for the next iteration. We modify the Rb block, by adding one deconvolutional layer to generate SR-scaled feature maps, without up-layer and without up-fusion-delivery, due to both blocks lacking the $Up(F_{Lnf}^{i})$ outputs. The result is listed in Table 2. Our proposed LNFSR-L outperformed all the comparing algorithms, in Table 2, illustrating the fusion structure affects the performance greatly. Although all the comparing algorithms are similar to three blocks in parallel, our proposed fusion structure (up-fusion-delivery block) can extract and deliver features to the right block, improving the fusion performance.

### 4.3. Quantitative Comparisons with State-of-the-Arts

In this section, we will give a comprehensive comparison of our LNFSR with other famous state-of-the-art SR algorithms, we choose SRCNN [2], VDSR [3], RCAN [21], EDSR [9], SRFBN [4] and CSNLN [5] as the state-of-the-art algorithms considered in this experiment, we also performed BiCubic up-sampling SR method as the baseline. We drop the noise or Gauss blurring tricks for all the chosen algorithms, due to different training skills will greatly affect the performance of different algorithms. Due to different algorithms in their original paper having different repeat times for each iteration, we fix it as 10 times to fairly compare. We perform the upscale factor range in [×2, ×3, ×4] for all the state-of-the-art SR algorithms. We also choose SRGAN [6] as the base-line of the GAN-based SR in scale ×4 (SRGAN focus on large scale). The results are listed in Table 3.

In Table 3, we reported the quantitative comparisons for scale factor ×2, ×3 and ×4. Where the BiCubic algorithm is considered the baseline of the SR algorithms and the SRCNN is considered the baseline of the deep learning-based SR algorithms. The CSNLN and our LNFSR both performed best, illustrating that self-attention-based algorithms outperform other state-of-the-art algorithms. Compared with the CSNLN, our LNFSR performed best in scale ×2 in all 4 famous standard benchmark datasets. In scale ×3 and ×4, our LNFSR outperformed the CSNLN algorithm in most of the standard benchmark datasets but underperformed in some special databases, especially in the scale ×4 on the Urban100 database, the reason is the Urban100 database focus on the constructions with much more far distance similar features, so CSNLN, which focus on non-local features, perform best in the scale ×4. We half the feedback iteration number of the base block, so we reduce the performance on non-local feedbackable features on large scale compared to the CSNLN, but our LNFSR outperforms the CSNLN (and other state-of-the-art algorithms) in most of the experiments.

### 4.4. Visualized Comparisons with State-of-the-Arts

**Figure 7:**
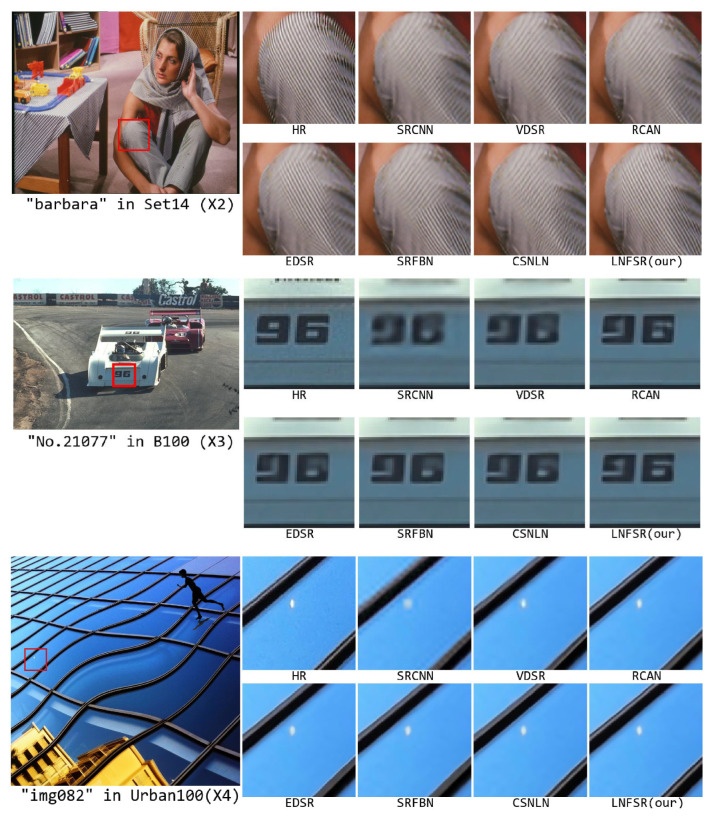
Visualized comparison on our LNFSR with other state-of-the-art Algorithms.

In this section, we will give a visualized analysis of our LNFSR with state-of-the-art as Figure 7. We take the same SR algorithms as Section 4.3. We drop the BiCubic to save space, and drop the SRGAN to get rid of confusion on its ’looks real’ SR image but lower PSNR.

The first picture in Figure 7 is the “barbara” which comes from the Set14 database under scale ×2. Our LNFSR generated the largest clean lines on her knee. Due to the feature being typically a long-distance similar picture, our LNFSR can achieve the best performance with fewer iterations (6 for our LNFSR, while 12 for CSNLN). The EDSR achieved the right SR images but a small clear area, while the SR images of the other algorithms (SRCNN, VDSR, RCAN, SRFBN, CSNLN) achieved the wrong line direction. Illustrating high-efficiency local feature extraction (EDSR) is critical for SR images to estimate the line direction, solo non-local feature extraction (RCAN, CSNLN) or local feature extraction (SRCNN, VDSR, SRFBN) may lead to being worth starting for SR image.

The second picture in Figure 7 is the No.21077 image which comes from the B100 database under scale ×3. Our LNFSR achieves commendable performance on the number “96”. The SR images of some algorithms (SRCNN, VDSR, EDSR) achieved a blurring number that is difficult to recognize, while the other SR images (RCAN, SRFBN, CSNLN) can achieve a plausible SR image but can be recognized, illustrating both local feature-based algorithm or non-local feature-based, which newly proposed, can achieve acceptable SR images. The RCAN, which is the classic non-local-based algorithm, achieved remarkable SR image outperforms other start-of-the-art algorithms, this means the number “96” image is easy for the non-local based algorithm, due to the long distance non-local feature help the algorithm to recognize the number “96” then generate the SR image.

The third picture in Figure 7 is the “img082” which comes from the Urban100 database under scale ×4. Our LNFSR achieves commendable performance, especially on the reflect lights. There are two lines of reflected light on the original HR image, and our LNFSR can exactly generate them. The SR images of local feature-based algorithms (SRCNN, VDSR, EDSR, SRFBN) failed to generate all the reflect lights, while the SR images of non-local feature-based algorithms (RCAN, CSNLN) can generate all the reflect lights but blurring reflect lights. This means the generation of the reflected lights needs local features and non-local features to cooperate with each other: non-local features can recognize the pattern of the reflected lights while local features refined them to generate a clear SR image.

From the visualized analysis above, we can see that the local and non-local features do not work alone, they need to cooperate with each other: non-local features help to recognize styles, while local features help to refine details. The CSNLN makes use of a wider range of attention than our purposed LNFSR (the DI = 18.11 for CSNLN while the DI = 12.80 for our LNFSR, Diffusion Index (DI) [22] is the newly proposed evaluation metric, A larger DI indicates more pixels are involved), but they did not fully make use of them, especially the local feature for refined SR image. Our LNFSR can extract both local and non-local features along with both repeatable and non-repeatable features, outperforming other state-of-the-art algorithms.

## 5. Conclusions

In this paper, we propose a new local and non-local features-based feedback network for SR (LNFSR). By separating the residual image into five different parts and choosing three different blocks to extract them, our proposed LNFSR can extract both local and non-local features along with both repeatable and non-repeatable features: (1) The traditional deep convolutional network block is used to extract the local non-feedbackable information directly and non-local non-feedbackable information (needs to cooperate with other blocks). (2) The dense skip-based feedback block is use to extract local feedbackable information. (3) The non-local self-attention block is used to extract non-local feedbackable information and the based LR feature information. We also introduced the feature up-fusion-delivery blocks to help the features be delivered to the right block at the end of each iteration. The experiments show that our proposed LNFSR can extract different kinds of feature maps by different blocks, and outperform other state-of-the-art algorithms. The limitation of our proposed LNFSR, which can be discovered in Figure 5, is the low usage of the DP blocks. So, our future work will focus on introducing the channel pruning [23] to further optimize our LNFSR.

## Figures and Tables

**Table 1:** The ablation study on the LNF block at ×2 scale on Set5. (The best performance is shown in **red**).

Algorithm	Only-DP	Only-FB	Only-CSNL	LNFSR-L
PSNR/SSIM	37.37/0.9581	37.57/0.9590	37.84/0.9600	37.90/0.9603

**Table 2:** The ablation study on the up-fusion-delivery block at ×2 scale on Set5. (The best performance is shown in **red**).

Algorithm	Without Up-Layer	Without Delivery	Without Up-Fusion-Delivery	LNFSR-L
PSNR/SSIM	37.80/0.9600	37.68/0.9594	37.66/0.9593	37.90/0.9603

**Table 3:** The performance (PSNR/SSIM) of the considered state-of-the-arts algorithms (the best performance is shown in **red** and the second-best performance is shown in **blue**).

Algorithm	Scale	Set5		Set14		B100		Urban100	
		PSNR	SSIM	PSNR	SSIM	PSNR	SSIM	PSNR	SSIM
BiCubic	×2	33.68	0.9303	32.23	0.8700	29.56	0.8435	26.87	0.8405
SRCNN [2]	×2	36.32	0.9519	32.24	0.9031	30.98	0.8824	28.96	0.8857
VDSR [3]	×2	37.46	0.9583	33.14	0.9131	31.91	0.8960	33.09	0.9170
RCAN [21]	×2	38.20	0.9612	33.93	0.9198	32.33	0.9015	32.83	0.9344
EDSR [9]	×2	38.11	0.9608	33.70	0.9182	32.23	0.9002	32.42	0.9308
SRFBN [4]	×2	38.03	0.9606	33.64	0.9177	33.22	0.9000	32.32	0.9304
CSNLN [5]	×2	38.17	0.9613	34.05	0.9213	32.32	0.9015	33.11	0.9371
LNFSR (our)	×2	38.22	0.9613	34.10	0.9229	32.35	0.9018	33.21	0.9377
BiCubic	×3	30.42	0.8688	27.58	0.7763	27.22	0.7395	24.46	0.7356
SRCNN[2]	×3	32.48	0.9046	29.07	0.8149	28.07	0.7768	25.79	0.7841
VDSR [3]	×3	33.73	0.9223	29.94	0.8343	28.84	0.7982	27.34	0.8326
RCAN [21]	×3	34.77	0.9298	30.58	0.8470	29.28	0.8095	28.95	0.8677
EDSR [9]	×3	34.69	0.9292	30.47	0.8455	29.22	0.8088	28.69	0.8644
SRFBN [4]	×3	34.57	0.9283	30.44	0.8442	29.17	0.8068	28.50	0.8594
CSNLN [5]	×3	34.63	0.9292	30.57	0.8470	29.26	0.8098	28.91	0.8682
LNFSR (our)	×3	34.72	0.9298	30.61	0.8468	29.26	0.8098	28.97	0.8677
BiCubic	×4	28.45	0.8110	26.04	0.7055	25.99	0.6692	23.15	0.6588
SRCNN [2]	×4	30.15	0.8531	27.20	0.7415	26.55	0.6985	24.05	0.7005
SRGAN [6]	×4	29.40	0.8472	26.02	0.7397	25.16	0.6688	-	-
VDSR [3]	×4	31.35	0.8825	28.16	0.7703	27.26	0.7244	25.28	0.7554
RCAN [21]	×4	32.51	0.8987	28.84	0.7876	27.73	0.7417	26.71	0.8058
EDSR [9]	×4	32.49	0.8985	28.81	0.7872	27.71	0.7409	26.58	0.8015
SRFBN [4]	×4	32.27	0.8963	28.69	0.7841	27.64	0.7379	26.35	0.7945
CSNLN [5]	×4	32.72	0.9008	28.97	0.7896	27.82	0.7451	27.34	0.8205
LNFSR (our)	×4	32.75	0.9013	28.97	0.7897	27.83	0.7451	27.33	0.8199

## Data Availability

Not applicable.

## Appendix A

For better reading this paper, we provide part of the acronyms and notations used in this paper in Table A1.

**Table A1:** The Acronyms and Notations.

Acronyms and Notations	Description
SISR	Single Image Super-Resolution
SR	Super-Resolution
HR	High-Resolution
LR	Low-Resolution
$I_{SR}$	Super-Resolution image
$I_{LR}$	Low-Resolution image
$I_{HR}$	High-Resolution image
L(Θ)	the loss function
FF(·)	the FF block
$F_{FF}$	the output features of the FF block
Lnf(·)	the LNF block
$F_{Lnf}^{i}$	the *i*-th iteration output of the LNF block
$F_{Lnf}$	the output features of the LNF block
Rb(·)	the Rb block
LNFSR(·)	our proposed LNFSR algorithm
DP[*i*] block	the *i*-th block of Deep Net (VDSR [3] in our paper)
FB block	the FeedBack block (SRFBN [4] in our paper)
CSNL block	the Cross-Scale Non-Local attention block
concat(·)	concat all the inputs
	on the feature dimension (second dimension)
Up(·)	Up layer block in the Up-Fusion-Delivery layer
Down(·)	down-scale the input to the LR scale
$DP_{dlr}\left(\cdot \right),FB_{dlr}\left(\cdot \right),CSNL_{dlr}\left(\cdot \right)$	the delivery layer of DP, FB and CSNL block
$DP_{up}\left(\cdot \right),FB_{up}\left(\cdot \right)$	the Up layer of DP and FB block
Fusion(·)	the fusion layer
